# Cryo-EM Structure of a Begomovirus Geminate Particle

**DOI:** 10.3390/ijms20071738

**Published:** 2019-04-08

**Authors:** Xiongbiao Xu, Qing Zhang, Jian Hong, Zhenghe Li, Xiaokang Zhang, Xueping Zhou

**Affiliations:** 1 State Key Laboratory of Rice Biology, Institute of Biotechnology, Zhejiang University, Hangzhou 310058, China; xiongbiaox1988@126.com (X.X.); lizh@zju.edu.cn (Z.L.); 2 Guangxi Key Laboratory of Medicinal Resources Conservation and Genetic Improvement, Guangxi Botanical Garden of Medicinal Plants, Nanning 530023, China; 3 Department of Biophysics, School of Medicine, Zhejiang University, Hangzhou 310058, China; 1618557@zju.edu.cn; 4 Center of Cryo Electron Microscopy, Zhejiang University, Hangzhou 310058, China; 5 Analysis Center of Agrobiology and Environmental Sciences, Zhejiang University, Hangzhou 310058, China; jhong@zju.edu.cn; 6 State Key Laboratory for Biology of Plant Diseases and Insect Pests, Institute of Plant Protection, Chinese Academy of Agricultural Sciences, Beijing 100193, China

**Keywords:** Cryo-EM, single particle analysis, asymmetric reconstruction, geminivirus, single capsid, geminate capsid

## Abstract

Tobacco curly shoot virus, a monopartite begomovirus associated with betasatellite, causes serious leaf curl diseases on tomato and tobacco in China. Using single-particle cryo-electron microscopy, we determined the structure of tobacco curly shoot virus (TbCSV) particle at 3.57 Å resolution and confirmed the characteristic geminate architecture with single-strand DNA bound to each coat protein (CP). The CP–CP and DNA–CP interactions, arranged in a CP–DNA–CP pattern at the interface, were partially observed. This suggests the genomic DNA plays an important role in forming a stable interface during assembly of the geminate particle.

## 1. Introduction

Geminiviruses are small, single-stranded plant DNA viruses that distribute widely in tropical and subtropical areas and cause many destructive crop diseases. The family *Geminiviridae* is divided into nine genera, namely *Mastrevirus*, *Becurtovirus*, *Begomovirus*, *Topocuvirus*, *Curtovirus*, *Eragrovirus*, *Turncurtovirus*, *Capulavirus*, and *Grablovirus*, according to their genome organization, biological properties, type of insect vectors, and host ranges [1]. The viral genome is composed of covalently closed circular single-stranded DNA (ssDNA) of about 2.5–3.2 kb for monopartite geminiviruses, and about 4.8–5.6 kb for bipartite geminiviruses. The virion morphology is unique amongst all known viruses in that two incomplete T = 1 icosahedra are fused together to form a twinned quasi-icosahedral virus particle that measures ~18 nm × 30 nm [2,3]. *Begomovirus* represents the largest genus in the family *Geminiviridae*, consisting of more than 320 species of either bipartite (DNA-A and DNA-B) or monopartite genomes (resembling DNA-A), and all of the members are transmitted by whitefly *Bemisia tabaci* [4]. Many monopartite begomoviruses are naturally associated with small circular alpha- and betasatellite DNAs of ~1.4 kb that are approximately half the size of viral genome. These satellite molecules are encapsidated by helper begomovirus-encoded coat proteins (CPs) and are transmitted by whitefly vectors [5]. Tobacco curly shoot virus (TbCSV) is a monopartite begomovirus naturally infecting nightshades such as tobacco (*Nicotiana tabacum*), tomato (*Lycopersicon esculentum*) [6], and pepper (*Capsicum frutescens*) [7] in Southeast China. Some TbCSV isolates have been found in association with betasatellites, but others seem to be able to dispense the need of the satellites to infect *Solanaceae* hosts [6].

Thus far, although the structures of African cassava mosaic virus (ACMV) and maize steak virus (MSV) have been characterized, the relative low resolution impedes detailed dissection of the virion structure and the interaction between viral genome and capsomers [8,9]. Recently, based on cryo-electron microscopy and image processing, Hipp et al. determined the ACMV virion structure with a resolution of 4.2 Å, built an atomic model for its capsid protein, and found a new pocket for possible DNA binding [3,8], A higher resolution (3.3 Å) 3D structure of *Ageratum* yellow vein virus (AYVV) was determined by using single-particle cryo-EM together with an atomic model, confirming the DNA binding-induced capsid conformational change [10]. Despite these progresses, detailed assembly mechanism of the geminate virion, especially how the genomic DNA is arranged across the interface of two single capsid, largely remains elusive. Here, we report a 3.57 Å Cryo-EM structure of TbCSV particles. The structure of TbCSV resembles general geminate capsid architecture and shows similar single-strand DNA binding sites as the AYVV, but reveals novel DNA–CP interactions at the assembly interface, which further completes the existing geminivirus particle assembly model.

## 2. Results and Discussions

### 2.1. Cryo-EM Structure of TbCSV

TbCSV particles were purified from infected plant leaves for Cryo-EM single particle analysis. To compensate the image blurring caused by the beam-induced movement of large particles during the course of long period exposure [11], dose fractioned movies were recorded with a 300 kV FEI Titan Krios G2 electron microscope equipped with a Gatan K2 summit direct electron detector camera. Motion-corrected dataset were then refined by removing low quality images, e.g., images with large accumulated motion, evidently massive ice-contamination or crystalline inside the view of image, and image contrast transfer function estimated correlation and estimated max resolution exceeded certain thresholds (Figure S6B), i.e. estimated CTF max resolution above 6 Å. Leaving micrographs with relative homogeneous particle distribution across the image (Figure S6A) revealed different views of the TbCSV particles (Figure S6E). During the particle picking process, a previous reconstruction was low pass filtered to a resolution of 30 Å to enable efficient and unbiased particle extraction. The extracted particle images were aligned with respect to each other and subjected to subsequent rounds of 2D reference free classification. Representative 2D class averages showed particles in both top view and side view (Figure S6C). The apparently varying length of the side view images was mainly due to the orientation of particles embedded in the vitrified ice, rather than variations in the general shape of the virions. D5 symmetry was enforced during subsequent image processing, and, after 3D-classification and several rounds of 3D auto refinement, a density map with 3.57 Å resolution at 0.143 Fourier shell correlation cut off criterion (gold standard) was determined (Figure S1).

**Figure 1:**
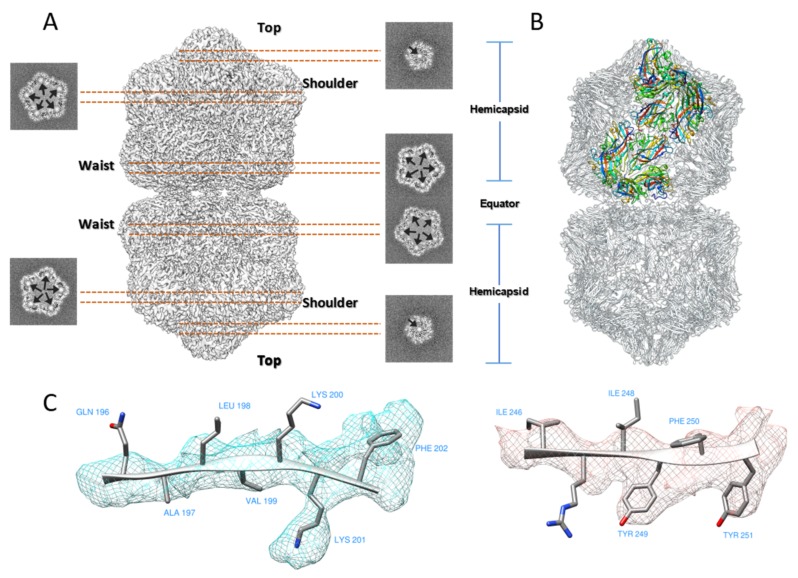
Cryo-EM structure of TbCSV Particles. (**A**) Surface representation of the D5-symmetrized EM map of TbCSV particle. From top to bottom, three types of different capsomers (top, shoulder, and waist) with five-fold or local five-fold symmetric capsomer are labeled. Horizontal slices cross the TbCSV map indicate density below the capsomers that might originate from DNA. (**B**) Complete model for all 110 CP subunits in the capsid, in which 11 CP subunits colored in rainbow indicate an asymmetric unit. (**C**) Representative areas with bulky sidechain densities used for model building.

Similar to the existing MSV [9], ACMV [3] and AYVV [10] structures, the final reconstruction of TbCSV showed a pentameric capsomer at the top layer, five capsomers with local five-fold symmetry at the shoulder layer, and five capsomers with quasi five-fold symmetry (Figure 1A). Each capsomer contained five CPs, thus there were 110 CPs totally in one geminate particle. The entire virion could also be seen as two quasi-icosahedral capsids joined together. The horizontal slices through the top, shoulder and waist layers showed extra densities inside the cavity below the capsomer densities (Figure 1A, arrows). These densities were similar to other published structures and might be derived from the genomic DNA. 

For model building, we chose AYVV model as the starting template because of its high sequence identity (Figure S5 and Table S2) compared to TbCSV among other published structures. Briefly, individual AYVV CP structures were rigidly fitted into the final reconstruction (Table S3), the amino acid sequence was manually adjusted, and the areas of the TbCSV EM map with bulky side-chain densities were used to facilitate the amino acid registration (Figure 1C). The final CP model was composed of 10 copies of asymmetric units (Figure 1B, in color), and each asymmetric unit covered 11 copies of CP (one in the top layer, five in the shoulder layer, and five in the waist layer). Five copies of the asymmetric unit formed a hemicapsid, which is a T = 1 particle without a penton of CP subunits.

**Figure 2:**
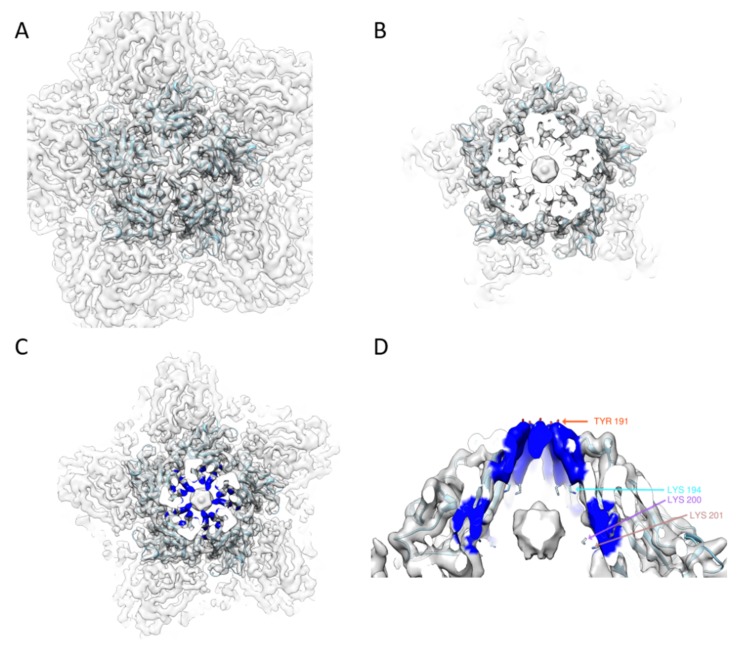
EM density around a five-fold capsomer: (**A**) superposition of the atomic model with the EM map of the TbCSV in one top capsomer; (**B**) the cut-through view of the top capsomer, with the possible DNA density located in the center; and (**C**,**D**) the top and side views around the center of the capsomer, the positively charged residues (colored in blue) forming a plug (K194) and a double layer ring surrounding the density attributed to DNAs.

From the atomic model, we found some features common to several different geminiviruses. In the center of a capsomer, five Tyr191 at the tip of five loops formed a hat and enclosed the particle (Figure 2A). Below the local five-fold symmetry axis (Figure 2B,C), there was a basket generated by five neighboring Lys194, Lys200 and Lys201 residues. This basket, together with the Y191 hat in the top, encapsulated the possible DNA density below each capsomer.

**Figure 3:**
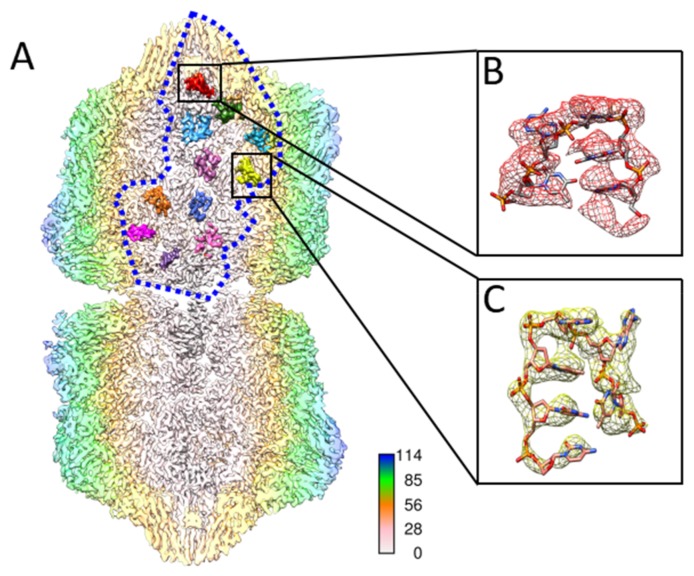
ssDNA densities in one asymmetric unit. (**A**) A total of 11 single-stranded DNA densities inside an asymmetric unit were separately colored in the rear half of TbCSV particle. The asymmetric unit is outlined by a blue dashed line. The TbCSV particle is presented as a longitudinal cross section, which is colored radially in the long axis. (**B**,**C**) Five and six nucleotides of DNA are modeled according to the density and zoomed in for details.

Furthermore, the TbCSV model resembled the arrangement of single-strand DNAlets observed in the AYVV model. Below 11 CPs in one asymmetric unit, there were 11 density regions inside the TbCSV reconstruction that were not occupied by the CP (Figure 3A). Depending on the area, up to six nucleotides could be built into the density unambiguously (Figure 3B,C). 

### 2.2. Asymmetric Analysis of TbCSV Dataset

To understand the arrangement of the genomic DNA inside the TbCSV virion, we carried out asymmetric reconstruction and 3D classification analysis of the TbCSV dataset. A large portion of the virus particles possessed high order of symmetry (dodecahedral, icosahedral and helical). Benefiting from this property, near-atomic and full-atomic resolutions are routinely achieved for structure determination of numerous viral capsids. However, inside the virion, structures of genomes and associated proteins inside the capsids are often missed, since the genome information may not follow the highly symmetric capsid information in the virus particle images [12,13,14]. To recover the missing genome information, the common practice of image processing is to be further relaxed to lower symmetries and generate another reconstruction without symmetry imposition. In this study, using cryoSPARC 2 ab-initio heterogenous reconstruction procedure with relaxed C1 symmetry, we generated several starting models from a set of TbCSV particle images, without any prior knowledge. Among these starting models, the one best resembled the characteristic twin particle feature and its associated particle images were chosen. After the cryoSPARC 2 homogeneous refinement procedure, the resolution was determined to 4.69 Å (Figure S2) using the Fourier shell correlation cut-off-criterion of 0.143 (gold standard), and the resulting angular distribution of the final 3D reconstruction showed a generally even angular orientation distribution, which permitted anisotropic resolution and excluded artifacts due to missing views. 

**Figure 4:**
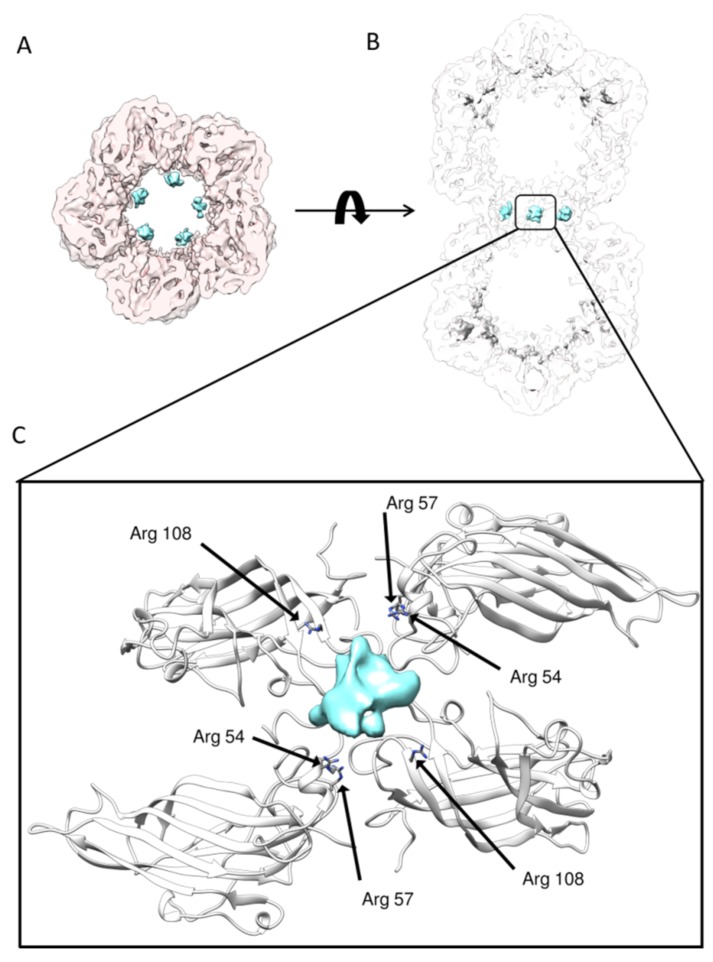
A novel DNA anchoring pocket at the interface: (**A**) extra densities (colored in cyan) are shown at the interface of two hemicapsids; (**B**) vertical slice view of the extra densities at the interface of two hemicapsids; and (**C**) detailed interactions between DNA and the positively charged residues from CP.

The overall shape of this asymmetric reconstruction was similar to the reconstruction with D5 symmetry enforced: three layers (top, shoulder and waist), large densities below each capsomer, and 11 ssDNA densities bound to each CP inside one asymmetric unit could be discerned from the map. This further verified the symmetry of previous obtained high resolution TbCSV reconstruction. However, due to the limited resolution attained, the exact model of ssDNA could not be derived directly from the asymmetric reconstruction. Besides, in the region where two hemicapsids joined together, five prominent densities were located surrounding the short channel formed by chain H and chain I of CP (Figure 4A,B). Based on the TbCSV atomic model, we analyzed this specific region of virion map in details. Generally, this basic amino acid-rich region consisted of Arg57/Arg60 from chain H and Arg108 from chain I immediately underneath the density. These positively-charged amino residues, together with their counterpart from the other hemicapsid, may serve as a DNA anchoring site and form a CP–ssDNA–CP pattern to support the interactions with DNA (Figure 4C). Moreover, these apparently asymmetric densities bound to CPs that exactly followed the five-fold symmetry, suggesting that the actual packaged genomic DNA extended through the two connected hemicapsids might spirally go through the interface in between. 

**Figure 5:**
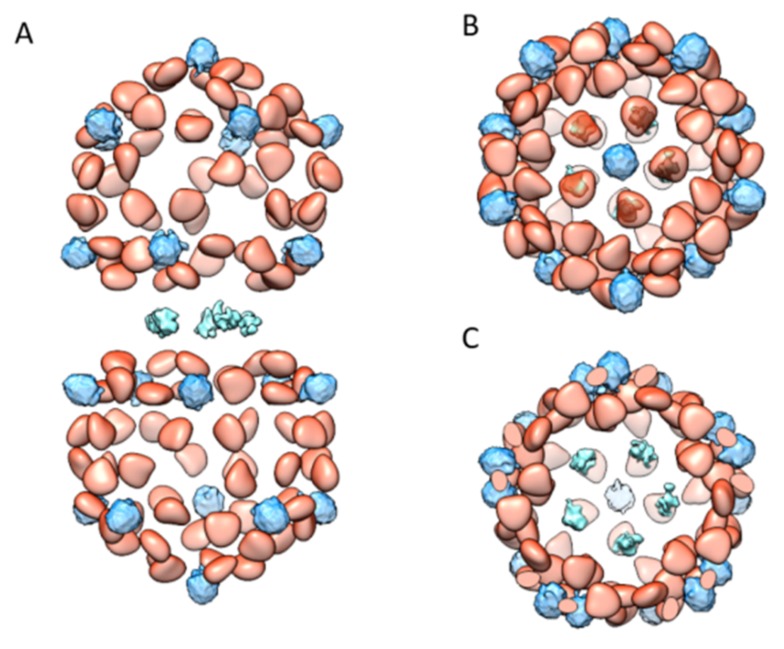
An overall model of known TbCSV DNA densities: (**A**) the side view of the TbCSV DNA densities; (**B**) the top view of the TbCSV DNA densities; and (**C**) the slice view of the TbCSV DNA densities at the interface between two hemicapsids. The model displays the known DNA densities across the entire geminate virion. The densities of ssDNA bound to the CPs are colored in orange, the DNA densities below the capsomers are colored in light blue, and the DNA densities across the interface of two hemicapsids are colored in cyan.

Combining all the observed DNA densities together, here we present an improved model that could better describe the location of genomic DNA (Figure 5). This model accounts for most of density seen inside the virion. These locations of DNA pockets, binding positions and anchoring sites in the TbCSV capsid followed certain symmetry. However, judging from the apparent density surface representations, the interaction partners did not always follow the strict symmetry of environment formed by the neighboring CPs. This observation suggests that the genomic DNA could spirally extend through the interior of the capsid and holds the possibility that genomic DNA do not have to occupy every single DNA pocket and binding position. These packaged DNA were situated inside most regions of the mature virion in an isolated manner, with the connections unresolved in our reconstructed density. The unresolved parts were most likely averaged out in image processing due to their flexible and plastic nature, and these flexible features were necessary for the assemble process. Thus, here we consider that these flexible DNA fragment might serve as the genomic packing signals [15]. To be more explicit, here we propose a model (Figure S4) of how the ssDNA extend through the interface between two hemicapsids. In this model, by occupying two neighboring DNA anchor sites, one strand of ssDNA spirally extends up into one hemicapsid and another strand of ssDNA spirally extends down into the other hemicapsid.

## 3. Materials and Methods 

### 3.1. Isolation and Purification of TbCSV

*N. benthamiana* plants infected with TbCSV alone or together with associated betasatellites (TbCSB) were harvested 4 weeks post-inoculation. Infected plant tissues (100 g) were ground in a homogenizer in 100-mL PB buffer (0.1 M Na_2_HPO_4_-NaH_2_PO_4_, pH 7.2) containing 2 mM EDTA and 10 mM Na_2_SO_3_. After thorough homogenization, the extract was filtered through four-layer muslin and stirred at 4 °C overnight in the presence of 1% (*v*/*v*) Triton X-100. Plant extract was clarified by addition of pre-chilled chloroform to final concentration of 10% (*v*/*v*), stirred for 15–30 min, and then subjected to low-speed centrifugation (8000 rpm) for 10 min in a Beckman JA-14 rotor. The aqueous phase was recovered and polyethylene glycol (PEG) 8000 and NaCl were added to final concentration of 12 g/100 mL and 0.2 M, respectively. After being stirred for 2–3 h at 4 °C, the extract was centrifuged for 15 min at 8000 rpm in a JA-14 rotor, and the pellet was resuspended in 1/10 of the original volume of resuspension buffer containing 0.1 M PB, 2 mM EDTA, and 1% Triton X-100. The suspension was stirred overnight at 4 °C, followed by centrifugation for 10 min at 10,000 rpm in a Beckman JA-25.5 rotor, and separated by ultracentrifugation for 1.5 h at 55,000 rpm in a Beckman Type 70 Ti rotor. The pellets were resuspended in 10 mL of resuspension buffer, followed by a low-speed centrifugation at 10,000 rpm for 10 min in a Beckman JA-14 rotor, and virions were precipitated by high-speed centrifugation in a Beckman Type 70 Ti rotor at 35,000 rpm for 2 h. The pellets were again resuspended in 4 mL resuspension buffer, stirred overnight at 4 °C, and then subjected to a low-speed centrifugation for 5 min at 6,000 rpm. The supernatant was then loaded on a 20% (w/v) sucrose cushion in 0.1 M PB buffer and centrifuged at 30,000 rpm for 3.5 h in a Beckman Type 70 Ti rotor. The recovered pellets were resuspended in 2 mL resuspension buffer and the suspension was layered on linear sucrose gradients (5–40%) prepared in 0.1 M PB buffer with 1 mM EDTA, followed by centrifugation at 20,000 rpm for 6 h in a Beckman SW 41 rotor. Virus particles were concentrated in the 15–25% sucrose gradients.

### 3.2. Grid Preparation and Cryo-EM Data Acquisition

Cryo-EM grids were prepared by applying TbCSV particles to 300 mesh grids (Quantifoil Micro Tools GmbH). Grids were glow-discharged for 60 s before applying the sample (easiGlow, Ted Pella). 2.5 μL sample was applied to the grids, blotted immediately using a FEI Vitrobot Mark IV plunge freeze (Thermo Fisher Co) device. Grids were frozen in liquid ethane cooled by liquid nitrogen at 100% relative humidity, and a chamber temperature of 20 °C. TbCSV data were collected on an FEI Titan Krios G2 (Center of Cryo Electron Microscopy, Zhejiang University) EM at 300 kV, using a total electron dose of 40 e-/Å^2^ and a magnification of 29,000×. A total of 2495 movies were recorded using the SerialEM [16] semi-automated acquisition software on a Gatan K2 summit direct electron detector in counting mode, with a final pixel size of 1.014 Å/pixel. Each exposure movie had a total exposure of 5 s and contained 20 frames.

### 3.3. Cryo-EM Image Processing

Image processing was performed using the RELION 2.1 [17,18]. Drift-corrected averages of each movie were created using MOTIONCOR2 [19], and the contrast transfer function of each movie was determined using Gctf [20]. A total of 60 images showing signs of significant aberration were manually identified and discarded. A previous reconstruction of TbCSV was low-pass filtered to 30 Å for automated particle picking using Gautomatch [21]. Particles were further classified using several rounds of both reference-free 2D classification and 3D classification (Figure S6C,D), with or without D5 symmetry imposed. A previous reconstruction was filtered to ~60 Å resolution for the starting model. After each round, the best classes/class were taken to the next step of classification. Post processing was employed to appropriately mask the model, to estimate and correct for the B-factor of the maps [22]. The final resolution was determined to be 3.57 Å using the “gold-standard” Fourier shell correlation (FSC = 0.143) criterion. For 3D asymmetric reconstruction and classification, all particles were used for ab initio 3D classification with cryoSPARC 2 [23] for C1 symmetry for two classes (Figure S7A,B). Tentative homogeneous refinements with C1 symmetry for these two classes generated two results: 10.55 Å for single capsid and 4.69 Å for geminate capsid. Local resolution (Figure S6C,D,F) was estimated using RESMAP [24].

### 3.4. Model Building

Sequence alignment was done by CLUSTAL W [25]. The sequence alignment figure was plotted by ENDScript [26]. Individual chains from the model of AYVV (6F2S) [10] were positioned within the TbCSV density map separately inside one asymmetric unit using rigid body fitting in UCSF Chimera [27]. the fitting correlations (Table S3) were assessed by the fit-in-map tool. Amino acid residues that did not fit into any density were removed and all remaining residues were changed to alanine using COOT [28]. The remaining backbone of the subunit was traced using COOT and the position of bulky amino acids was used to manually add the TbCSV CP sequence. DNA nucleotides were manually built into the density by COOT. The resulting model of 11 subunits that covered one asymmetric unit was symmetrized using Chimera. This asymmetric unit was subsequently refined using the “real space refinement tool” in COOT followed by Phenix [29]. Model related statistics are summarized in Table S1. UCSF Chimer [27] was used to render the figures.

## 4. Conclusions

In this study, we purified the TbCSV virion from infected *N. benthamiana*, and determined the 3D structure of TbCSV virion to 3.57 Å resolution. The structure revealed 22 encapsidated DNA interaction baskets and 11 ssDNA densities bind to each CP in 11 asymmetric units. In addition, we located five extra densities at interface of two hemicapsids of the TbCSV cryoEM density map by using asymmetric reconstruction and 3D classification. Remarkably, the five densities located in the interface of the two hemicapsids of our cryoEM asymmetric structure were different from each other; the usage of the asymmetric reconstruction and 3D classification was considered crucial to identify these subtle but essential differences among them. This observation implies that the single-stranded, circular genomic DNA may spirally extend through the narrowed cavities formed by the 20-copy twisted CPs at the two waists of the geminate virion. To better understand the structure of the TbCSV virion particles, especially the environment at the interface of two hemicapsids, we built an atomic model based on our cryo-EM 3D map by utilizing the bulky side chain densities as the registration marks. In this model, three basic amino acids (Arg57 and Arg60 in Chain H, Arg108 in Chain I) were identified in the vicinity of the density at the interface of two hemicapsids. These residues, together with their counterpart in the other hemicapsid, formed a CP–ssDNA–CP pattern. In this arrangement, this evolutionarily conserved (Figure S3) positively charged region may serve as a DNA anchoring site when the whole genomic DNA passes from one hemicapsid to the other. Additionally, ssDNA binds to this region and may support and extend the CP to form interface scaffold. Here, to supplement the proposed model [10] of geminate particle assembly originated from the AYVV atomic model, we suggest an assembly mechanism whereby the genomic DNA first serves as the scaffold in the beginning, then individual CPs bind to the corresponding regions of circular ssDNA and undergo several confrontational changes. Consequently, the CPs intermediates form the interface assembly in the middle of the virion. In summary, our study indicates that the genomic DNA plays a crucial role in the interface of two single capsids and further enriches the knowledge of the assembly mechanism of geminivirus particles in general.

## Supplementary Materials

The following are available online at https://www.mdpi.com/1422-0067/20/7/1738/s1, Figure S1: Gold Standard FSC curve of D5-averaged reconstruction of TbCSV, Figure S2: Gold Standard FSC curve of asymmetric reconstruction of TbCSV, Figure S3: Sequence alignment of CPs from several Geminivirus, Figure S4: A possible genomic DNA arrangement: (A) front view; and (B) tear view of possible genomic DNA arrangement of Geminivirus, Figure S5: Sequence alignment of CP of TbCSV with ACMV, MSV and AYVV, Figure S6: (A) A typical Cryo-EM micrograph of TbCSV. The scale bar represents 20 nm. (B) Parameters of contrast transfer function were determined with GCTF. Most micrographs have resolution beyond 3 Å. (C) 2D Classification of the TbCSV particles, where red boxes are chosen classes. (D) 3D Classification of the TbCSV particles, where red boxes are chosen classes. (E) Euler angle distribution. All orientations are covered in the dataset, but the view from the bottom is somewhat preferred. (F) Local resolution of the electron density map, Figure S7: (A) Ab-initio asymmetric starting models; (B) Euler angle distribution of the asymmetric reconstruction; (C) local resolution of the electron density map of asymmetric reconstruction at the interface; and (D) local resolution of the electron density map of asymmetric reconstruction of the capsid, Table S1: Parameters used for Cryo-EM image collection and model refinement, Table S2: Coat protein sequence percent identity matrix among MSV, ACMK-K, TbCSV-Y35 and AYVV, Table S3: Correlations between AYVV model (6F2S) derived map and TbCSV map.

## Abbreviations

3D	Three-dimensional
ACMV	African cassava mosaic virus
AYVV	Ageratum yellow vein virus
CP	Coat protein
EM	Electron microscopy
MSV	Maize streak virus
ssDNA	Single-stranded DNA
TbCSV	Tobacco curly shoot virus
