# Supplementary material for: Cryo-EM Structure of a Begomovirus Geminate Particle
Source: Int J Mol Sci. 2019 Apr 8;20(7):1738. doi: 10.3390/ijms20071738 (PMC6480954; doi:10.3390/ijms20071738)
Supplement: Supplementary file 1 [file ijms-20-01738-s001.pdf]

Supplementary Material

Cryo-EM structure of a Begomovirus Geminate Particle

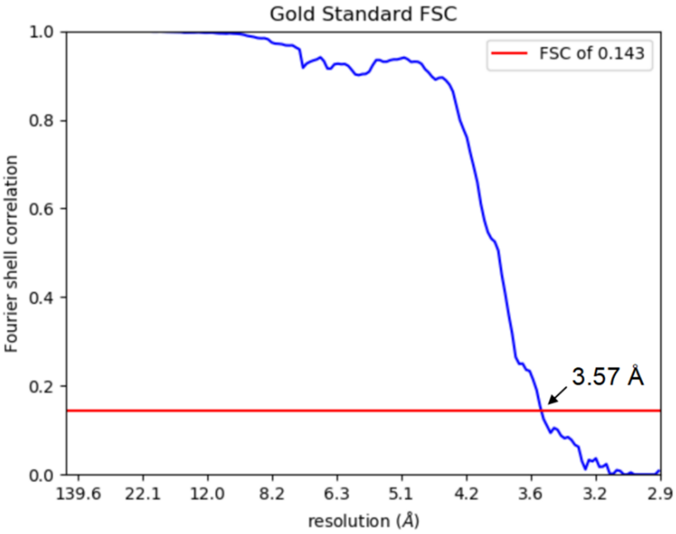

Figure S1. Gold Standard FSC curve of D5-averaged reconstruction of TbCSV.

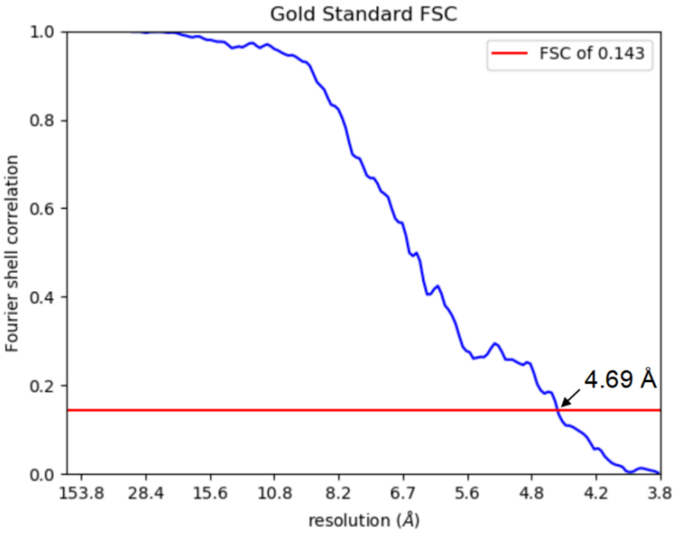

Figure S2. Gold Standard FSC curve of asymmetric reconstruction of TbCSV.

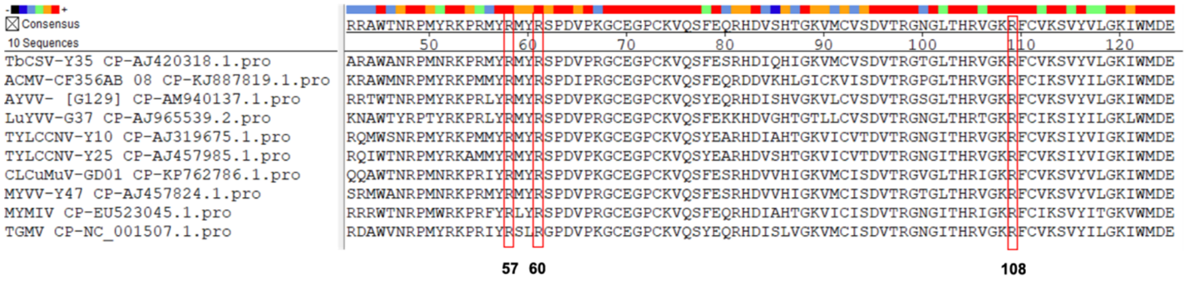

Figure S3. Sequence alignment of CPs from several Geminivirus.

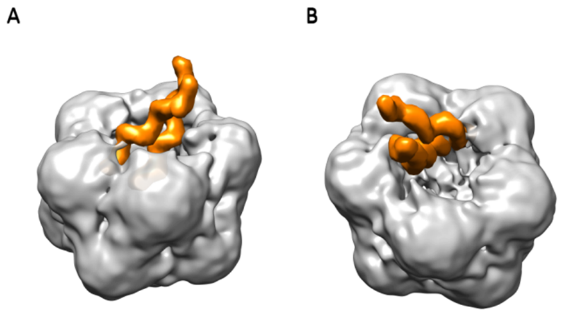

Figure S4. A possible genomic DNA arrangement: (**A**) front view; and (**B**) tear view of possible genomic DNA arrangement of Geminivirus.

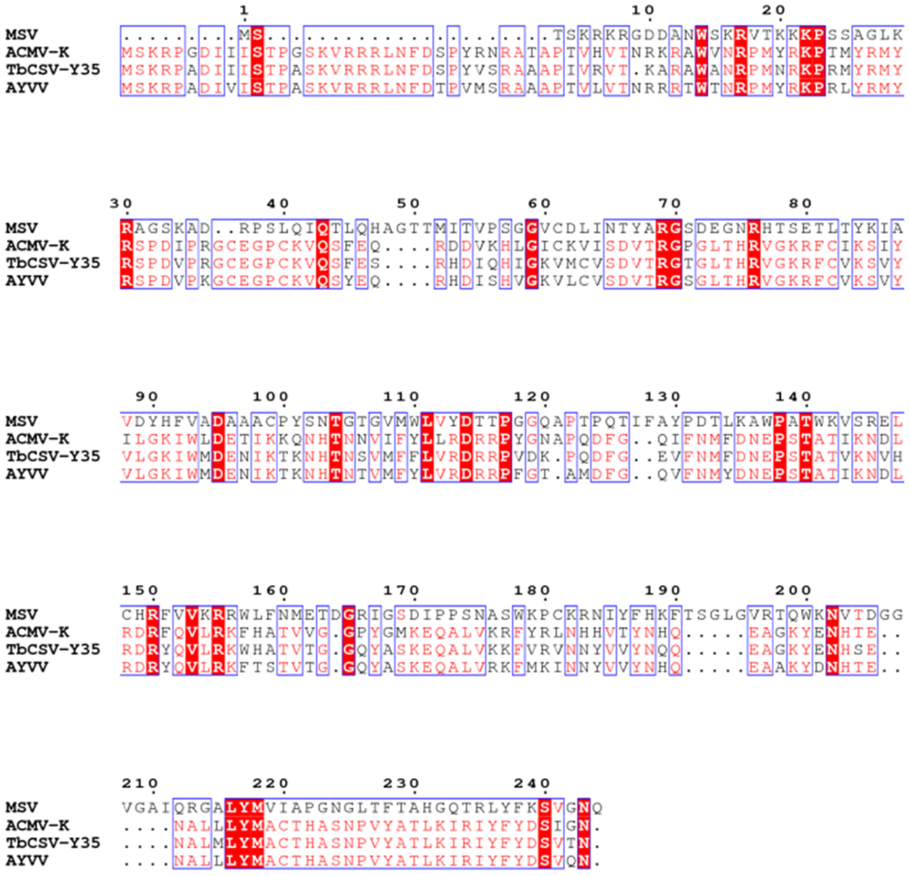

Figure S5. Sequence alignment of CP of TbCSV with ACMV, MSV and AYVV.

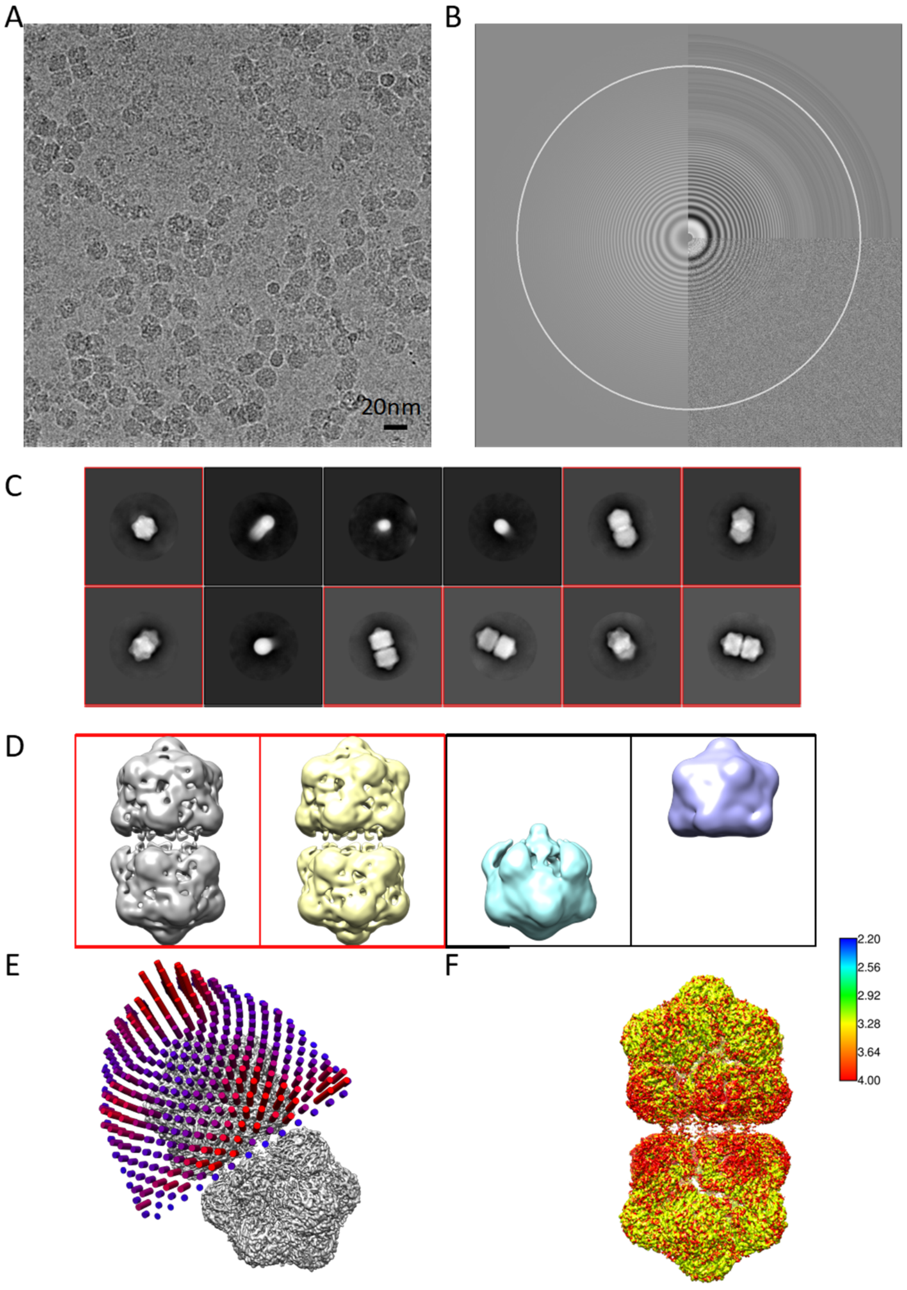

Figure S6. (**A**) A typical Cryo-EM micrograph of TbCSV. The scale bar represents 20 nm. (**B**) Parameters of contrast transfer function were determined with GCTF. Most micrographs have resolution beyond 3 Å. (**C**) 2D Classification of the TbCSV particles, where red boxes are chosen classes. (**D**) 3D Classification of the TbCSV particles, where red boxes are chosen classes. (**E**) Euler angle distribution. All orientations are covered in the dataset, but the view from the bottom is somewhat preferred. (**F**) Local resolution of the electron density map.

.

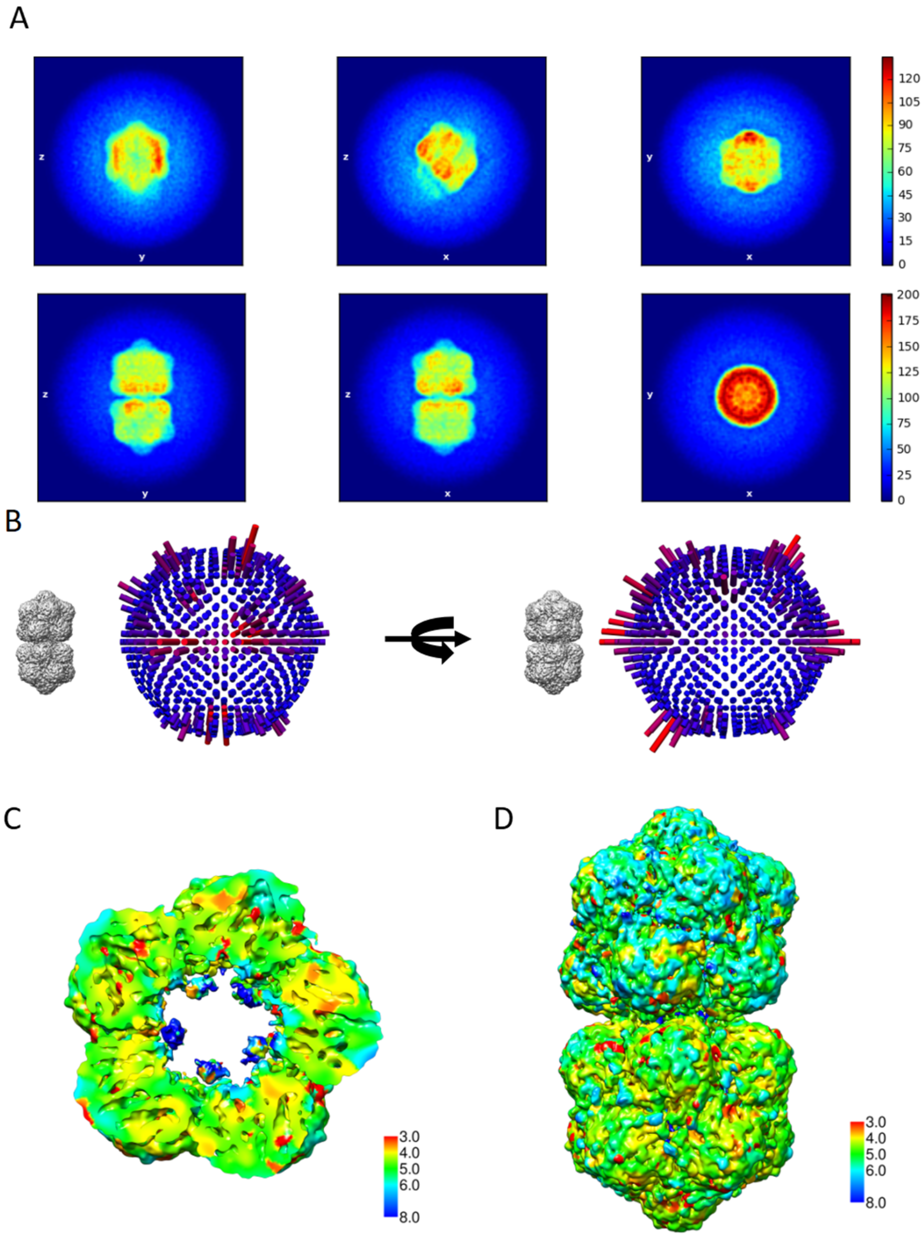

Figure S7. (**A**) Ab-initio asymmetric starting models; (**B**) Euler angle distribution of the asymmetric reconstruction; (**C**) local resolution of the electron density map of asymmetric reconstruction at the interface; and (**D**) local resolution of the electron density map of asymmetric reconstruction of the capsid.

Table S1. Parameters used for Cryo-EM image collection and model refinement.

Parameter	Value
Pixel size, Å	1.014
Defocus range, µm	1-3
Acceleration voltage, kV	300
Dose, e/Å2	40
Movies	2495
Used Movies	2435
Extracted particles	102109
Particles for 3D final maps	53890
Symmetry for final maps	D5
Final resolution, Å	3.57
RMSD bond lengths, Å	0.008
RMSD bond angles, °	0.959
Avg B factor	117.2
MolProbity score	1.92
Clash score	7.92
Good rotamers, %	98.51
Ramachandran favored, %	94.75
Ramachandran allowed, %	4.88
Ramachandran outliers, %	0.37

Table S2. Coat protein sequence percent identity matrix among MSV, ACMK-K, TbCSV-Y35 and AYVV.

	**MSV**	**ACMV-K**	**TbCSV-Y35**	**AYVV**
MSV	100	17.78	16.14	16.52
ACMV-K	17.78	100	76.56	75.88
TbCSV-Y35	16.14	76.56	100	82.81
AYVV	16.52	75.88	82.81	100

Table S3. Correlations between AYVV model (6F2S) derived map and TbCSV map.

**6F2S**	**Correlation**
Chain A	0.856
Chain B	0.855
Chain C	0.855
Chain D	0.853
Chain E	0.854
Chain F	0.856
Chain G	0.848
Chain H	0.838
Chain I	0.841
Chain J	0.846
Chain K	0.847
